# NO-dependent cGMP signalling in neurons and astrocytes

**DOI:** 10.1186/2050-6511-16-S1-A55

**Published:** 2015-09-02

**Authors:** Jan Giesen, Ernst-Martin Füchtbauer, Doris Koesling, Michael Russwurm

**Affiliations:** 1 Institute of Pharmacology and Toxicology, Ruhr-University-Bochum, Bochum, Germany; 2 Department of Molecular Biology and Genetics - Molecular Cell and Developmental Biology, Aarhus University, Denmark

## 

In the central nervous system, NO-dependent cGMP signalling is associated with many different developmental processes and brain functions, including neurogenesis, neuronal development and memory formation. The NO/cGMP signalling cascade is involved in regulating pre- and postsynaptic action by facilitating glutamate release and increasing NMDAR-mediated currents, respectively. These different actions of NO/cGMP have been ascribed to the distinct subcellular localisation of the two known NO-GCs.

Immunohistochemical studies indicate a presynaptic localisation of NO-GC1, whereas NO-GC2 is found at the postsynaptic site. Both NO-GC isoforms are required for LTP, as knock-out mice of either one of the NO-sensitive GCs show impaired induction of hippocampal and cortical LTP.

FRET-based cGMP indicators (cGi) are an important tool to analyze the temporal resolution of cGMP signals in living cells. Therefore, a transgenic mouse was generated which stably and ubiquitously expresses a cGMP indicator (cGi500). Confocal laser scanning microscopy was used to monitor cGMP signalling in primary cells from cGi500 knock-in mice.

Fast and reversible NO-induced cGMP signals were detectable in neurons and astrocytes. Maximal GSNO-induced cGMP responses were similar in both cell types. Cyclic GMP signals in neurons were elicited by approximately ten fold lower GSNO concentrations compared to astrocytes. Whether lower responses of astrocytes to exogenous applied NO could be due to lower amount of cGMP-generating enzymes or different expression pattern of PDEs will be further studied in detail.

